# Experimental outgassing of toxic chemicals to simulate the characteristics of hazards tainting globally shipped products

**DOI:** 10.1371/journal.pone.0177363

**Published:** 2017-05-17

**Authors:** Lygia Therese Budnik, Nadine Austel, Sabrina Gadau, Stefan Kloth, Jens Schubert, Harald Jungnickel, Andreas Luch

**Affiliations:** 1 Occupational Toxicology and Immunology Unit, Institute for Occupational and Maritime Medicine (ZfAM), University Medical Center Hamburg-Eppendorf, University of Hamburg, Hamburg, Germany; 2 Department of Chemical and Product Safety, German Federal Institute for Risk Assessment (BfR), Berlin, Germany; Northwest Fisheries Science Center, UNITED STATES

## Abstract

Ambient monitoring analyses may identify potential new public health hazards such as residual levels of fumigants and industrial chemicals off gassing from products and goods shipped globally. We analyzed container air with gas chromatography coupled to mass spectrometry (TD-2D-GC-MS/FPD) and assessed whether the concentration of the volatiles benzene and 1,2-dichloroethane exceeded recommended exposure limits (REL). Products were taken from transport containers and analyzed for outgassing of volatiles. Furthermore, experimental outgassing was performed on packaging materials and textiles, to simulate the hazards tainting from globally shipped goods. The mean amounts of benzene in analyzed container air were 698-fold higher, and those of ethylene dichloride were 4.5-fold higher than the corresponding REL. More than 90% of all containers struck with toluene residues higher than its REL. For 1,2-dichloroethane 53% of containers, transporting shoes exceeded the REL. In standardized experimental fumigation of various products, outgassing of 1,2-dichloroethane under controlled laboratory conditions took up to several months. Globally produced transported products tainted with toxic industrial chemicals may contribute to the mixture of volatiles in indoor air as they are likely to emit for a long period. These results need to be taken into account for further evaluation of safety standards applying to workers and consumers.

## Introduction

With the globalized production and trade, most small and large companies import production parts, raw materials and goods from overseas and consumers place their individual orders anywhere in the world [1, 2]. To ensure preservation and quality of these goods, chemical agents (e.g. methyl bromide) for pest control or to stop the introduction of non-indigenous species are added either to the shipped items or to transport units [3, 4]. Treatment of materials with volatile chemical agents referred to as fumigation is regulated by the international standards of the UN Food and Agriculture Organization (FAO) for phytosanitary measures (ISPM 15). This applies to the possible translocation of pests in vehicles, ships, aircrafts, containers and all sorts of storage items and areas as well as to packaging materials designed for overseas transportation[5]. ISPM 15 is especially important for containers, which include goods packed with wooden material (e.g. euro pallets); they have to be treated either with methyl bromide (bromomethane) or heat.

Residual levels of fumigants and industrial chemicals outgassing from fright containers may constitute possible health risks. Also goods and packaging materials may emit harmful volatile inorganic and organic compounds (VICs and VOCs) that stayed in the product after the production process and that might accumulate in the air inside the closed container. Workers exposure to residual chemicals at workplaces dealing with container unloading or product storage areas was reported before [6–11]. In about 70% of containers arriving in European and overseas harbors residual chemicals were detected [10, 12–16]. It became clear that beside the fumigants the container, packaging materials and therein transported items could be tainted with various industrial chemicals like toluene, dichloromethane, benzene and ethylene dichloride (production residuals, packaging materials, cleaning activities or various chemical formulations improving the fumigant quality or its fire resistance)[17]. After arriving in harbors, closed transport units are relocated to often far-away cities or areas before they are unloaded and opened. Then the goods are distributed and used by workers, bystanders and consumers, who are often unaware of prior fumigation processes[8]. Although evidence is emerging that products tainted with industrial chemicals may release these substances for rather long periods after accessing, there is only limited data on outgassing characteristics of diverse chemicals, which may allow proper health-based risk assessment. Toxic industrial chemicals, especially toluene, dichloromethane, benzene and ethylene dichloride may exert adverse health effects, from acute airway irritation to cancer[18–23]. In practical terms, rewards from understanding how toxic industrial chemicals interact with products are large. Babies, children, the elderly and health compromised individuals are the most vulnerable people in our society and even small daily doses of exposure to harmful chemicals in the air and from outgassing products might cause irreversible damage to their health. Small scale releases of toxic chemicals are common in the industrialized world, but low dose long-term exposure scenarios and their impact on human health are only rarely investigated. There is little data available about indoor home low dose exposure of consumers to chemicals. The most valuable data source is the RIOPA study evaluating exposures against mixtures of VOCs[22, 24, 25]. Focusing on non-smoker homes, the data show that indoor sources generally contribute to the majority of VOC exposure for most people and that concentrations of indoor VOCs typically exceeded outdoor levels (e.g. indoor vs outdoor ratio for toluene of 4.6). Unfortunately, although the study provides valuable information on outdoor, indoor and personal exposures, the RIOPA study focused only on odorant and cleaning-related VOCs like chloroform, 1,4-dichlorobenzene and styrene in its mixtures analyses[22]. Nevertheless the study identified median benzene levels of 1.3 μg/m3 (75%: 4.0 μg/m^3^; 90%: 9.5 μg/m^3^; maximum benzene air level: 90 μg/m^3^) and median toluene levels of 10 μg/m^3^ (75%: 22 μg/m^3^; 90%: 49 μg/m^3^; maximum toluene air levels: 368 μg/m^3^). Raw et al. [26]focused on potential determinants of exposure in 876 homes in England showing similar values of maximum benzene concentrations of 93.5 μg/m^3^ (geometric mean of 3 μg/m^3^) and much higher maximum toluene concentrations of 1,783 μg/m^3^ (geometric mean 15.1 μg/m^3^). The values were significantly higher in the winter period, indicating the importance of room aeration. No methylene chloride or ethylene dichloride was measured in either study. Considering the data from the RIOPA and TEAM studies, Weisel [27]looked into the association between indoor ambient exposure and asthma. The author stressed the importance of target population analysis with respect to adverse health endpoints. Bolden and co-workers identified epidemiological studies assessing the non-cancer health impacts of ambient level benzene, toluene and other BTEX exposure[28]. Focusing on endocrine disrupting effects, the authors have shown that low level exposure to BTEX may induce sperm abnormalities, reduced fetal growth, cardiovascular disease, respiratory dysfunction, asthma, sensitization to common antigens, and more. Health effects were observed at exposure concentrations that were in many cases orders of magnitude below the U.S. EPA reference concentrations (i.e., safe daily exposure level)[28].

The aim of our study was to provide experimental data allowing future risk assessment of possible health risks from products tainted with fumigants and industrial chemicals. Such understanding will increase our ability to control and prevent exposures.

In this study, we have screened the air of 2,027 import containers for VICs and VOCs. We tested whether the concentration of the volatiles exceeded recommended exposure limits and if there is a relationship of the transported goods and the VIC/VOCs measured. Furthermore, goods from suspicious containers were analyzed for outgassing from the transported goods. For a better understanding of the desorption behavior of fumigants from consumer products, we conducted fumigation experiments and analyzed the outgassing of classical fumigants (phosphine, methyl bromide) and ethylene dichloride (1,2-dichloroethane) from packaging materials, textiles and food for detailed time course analyses.

## Materials and methods

### Screening of container air samples

The requirement for an effective monitoring of residual fumigant contamination in the air of imported freight containers led us to develop and validate a mass spectrometry method based on mass spectrometry combined with thermal desorption gas chromatography (TD-GC/MSD), allowing the simultaneous determination of major fumigants such as methyl bromide, sulfuryl fluoride (sulfuryl difluoride), methyl iodide (iodomethane), propylene dichloride (1,2-dichloropropane), ethylene dichloride (1,2-dichloroethane), chloropicrin (trichloronitromethane), and the toxic industrial solvents benzene, toluene and carbon disulfide[13]. The method was also developed to simultaneously detect phosphine along with VOCs in container air samples using a thermal desorption system coupled to a two dimensional gas chromatograph with mass spectrometric and flame photometric detection (TD-2D-GC-MS/FPD). By incorporating simultaneous collection of selected ion monitoring (SIM) and SCAN data, single analysis was previously found sufficient for qualitative screening and quantification of all target compounds[29].

The container sampling was permitted and supported by the Federal Customs Office in Hamburg.Air samples were taken using a tubular steel lance pushed through the container door seal and a silicon tube connected to a Tedlar^®^ sample bag in the Vacu-Case™ vacuum pump (both Analyt MTC, Mühlheim, Germany). 1 L of air was taken from each of the 2,027 containers arriving at the Customs Office in the port of Hamburg, Germany. A certified test mixture of 39 compounds in the gas phase was purchased from Scott (Scott Specialty Gasses, PA, USA). Additionally, certified standard gases of methyl bromide (bromomethane), phosphine and sulfuryl fluoride were obtained from Linde (Linde AG, Gases Division Germany, Pullach, Germany). Analytical grade liquid compounds benzene, carbon disulfide, 1,2-dichloroethane, 1,2-dichloropropane, dichloromethane, ethyl benzene, iodomethane, toluene, tetrachloromethane and trichloronitromethane were purchased from Fluka Analytical (Fluka Analytical/Sigma-Aldrich Switzerland, Buchs, Switzerland). The gas chromatograph was run in constant pressure mode using the *Deans* column switch. Helium 5.0 was used as carrier gas and was further purified using a helium gas filter (Supelcarb HC, Supelco/Sigma-Aldrich, Sigma-Aldrich Switzerland, Buchs, Switzerland) to trap oxygen, water and hydrocarbons as described earlier. Columns were chosen to separate phosphine and sulfuryl fluoride from the VOCs on the first column and to separate phosphine from sulfuryl fluoride on the second one. Phosphine and sulfuryl fluoride were the first compounds of interest to elute from column #1. The corresponding peak was switched to the second column where the two compounds were separated and eluted to the FPD in phosphorus mode. All other compounds eluting from the first column were analyzed by MS in scan mode for compound identification and in SIM mode for quantification. All VOCs were well separated in the first dimension on the HP-1MS column, while phosphine and sulfuryl fluoride were separated sufficiently in the second dimension on the PLOT column. More details on the method were published elsewhere[29]. Limits of detection and quantification were derived from low concentration standard curves by appropriate equations:

$$LOD=s_{x_{0}}\cdot t_{f,\alpha }\cdot \sqrt{\frac{1}{N_{a}}+\frac{1}{N_{c}}+\frac{{\bar{x}}^{2}}{Q_{x}}}$$

$$LOQ=k\cdot s_{x_{0}}\cdot t_{f,\alpha }\cdot \sqrt{\frac{1}{N_{a}}+\frac{1}{N_{c}}+\frac{{\left(k\cdot LOD-\bar{x}\right)}^{2}}{Q_{x}}}$$

$$\begin{array}{l} LOD=\text{limit of detection}\\ \text{LOQ}=\text{limit of quantification}\\ {\text{s}}_{{\text{x}}_{0}}=\text{standard deviation}\\ {\text{t}}_{\text{f},\alpha }=\text{factor of t distribution}\\ {\text{N}}_{\text{a}}=\text{number of measurements}\\ {\text{N}}_{\text{c}}=\text{number of calibration points}\\ \bar{x}=\text{mean of concentrations}\\ {\text{Q}}_{\text{x}}=\text{summ of square deviations}\\ \text{x}=\text{concentration} \end{array}$$

Note the conversion factors from μL/m^3^ (μL/m^3^ = ppb) to μg/m^3^ for the target compounds at 23°C (laboratory temperature): phosphine: 1.4; dichloromethane: 3.50; methyl bromide/ bromomethane: 3.91; 1,2-dichloroethane: 4.07; toluene: 3.79; and benzene: 3.21.

### Outgassing of container-origin products

Children toys (n = 23), shoes and socks (n = 15) were taken out from shipping containers and transferred to a desorption chamber. After 24 h an air sample was taken and analyzed by TD-2D-GC-MS/FPD (see above). The samples have been taken from containers, which have exceeded the recommended exposure limit for one of the analyzed toxic industrial chemicals (ethylene dichloride, methylene chloride or toluene). The products (n = 38, not randomized) were placed in an evaporating chamber at room temperature (21^°^C, 30% relative humidity) for 24 h. (The 1.24 m^3^ outgas volume with continuous ventilation of 1.14 m^3^/h/m^2^ provides a good model for a small room of about 11 m^2^, as based on the methods for VOC emissions from construction products, European Commission EUR 17334-Rep no.18), Then the TD-GC/MSD analysis of the residual outgassing chemicals was performed (see above).

### Experimental outgassing

We decided to perform standardized experimental fumigation of various products to look into the outgassing kinetics under controlled laboratory conditions. Further we aimed to analyze whether fumigation with these chemicals may affect the properties of the products. In an outset, we have chosen to fumigate socks and packing material (wrapping paper) to elaborate the possible differences in its outgassing patterns. To investigate the desorption behavior of fumigants from consumer products in a detailed time course, we fumigated wrapping paper (80 g/m² 100% cellulose) and nylon socks (85% polyamide and 15% elastane) with 100 ppm phosphine, methyl bromide or 1,2-dichloroethane, for 72 h in a fumigation chamber of 4 L volume (3 independent replicates). After fumigation samples were transferred to a desorption chamber (53 L). At consecutive days, air samples were collected repeatedly from the side of the chamber (see digital abstract) with the help of a gas jumbo syringe (with 1 L volume). After each sampling, the chamber had been ventilated completely with fresh air to simulate natural conditions at a storage room or a consumer home. This procedure has been repeated on the following sampling days till the concentration of fumigants in the air samples reached the detection limit. Air samples (transferred from the gas jumbo syringe into tedlar bags) were analyzed by TD-2D-GC-MS/FPD (see above).

Surface analyses using time-of-flight secondary ion mass spectrometry (Tof-SIMS) were performed to investigate, whether the product surfaces may be especially susceptible for the absorption/desorption of toxic gases (data not shown). Fumigated samples of wrapping paper were analyzed by Tof-SIMS to look into interactions of the fumigant with molecules of the consumer product to reflect the adhesion of the fumigants to the product surface. For each sample (n = 3 independent fumigations, see above), 1 cm² have been cut out on dry ice and a depth profile and a surface scan in positive and negative mode have been taken.

### Data analysis

For the interpretation of the results, independent, international scientifically based Reference Exposure Levels (RELs) were used. As limit values the chronic RELs released by the US Office of Environmental Health Hazard Assessment, OEHHA were applied[30]. The REL values were as follows: 400 **μ**g/m^3^ (102 **μ**L/m^3^, ppb) for methylene chloride (dichloromethane), 400 **μ**g/m^3^ (98 **μ**L/m^3^, ppb) for ethylene dichloride (1,2-dichloroethane), 300 **μ**g/m^3^ (79 **μ**L/m^3^, ppb) for toluene and 3 **μ**g/m^3^ (0.98 **μ**L/m^3^, ppb) for benzene. These RELs are derived from the most sensitive non-cancer health effect reported in medical and toxicological literature for a particular target tissue (either in the nervous, respiratory, cardiovascular or alimentary system or for developmental processes). The values are designed to protect those individuals who live or work in the vicinity of emission sources and who are continuously exposed to these substances.

Data evaluation was performed using descriptive statistics with univariate analysis. Subgroups were formed according to the major categories of the type of goods or contents as declared to the customs authorities and the type of contaminating chemical detected during the course of investigation. These were further subdivided into subsets for data analysis. The statistical analysis was performed with Graph PAD 6.05.

## Results

### Analysis: Presumably carcinogenic chemicals in container air vs. transported goods

**Fig 1:**
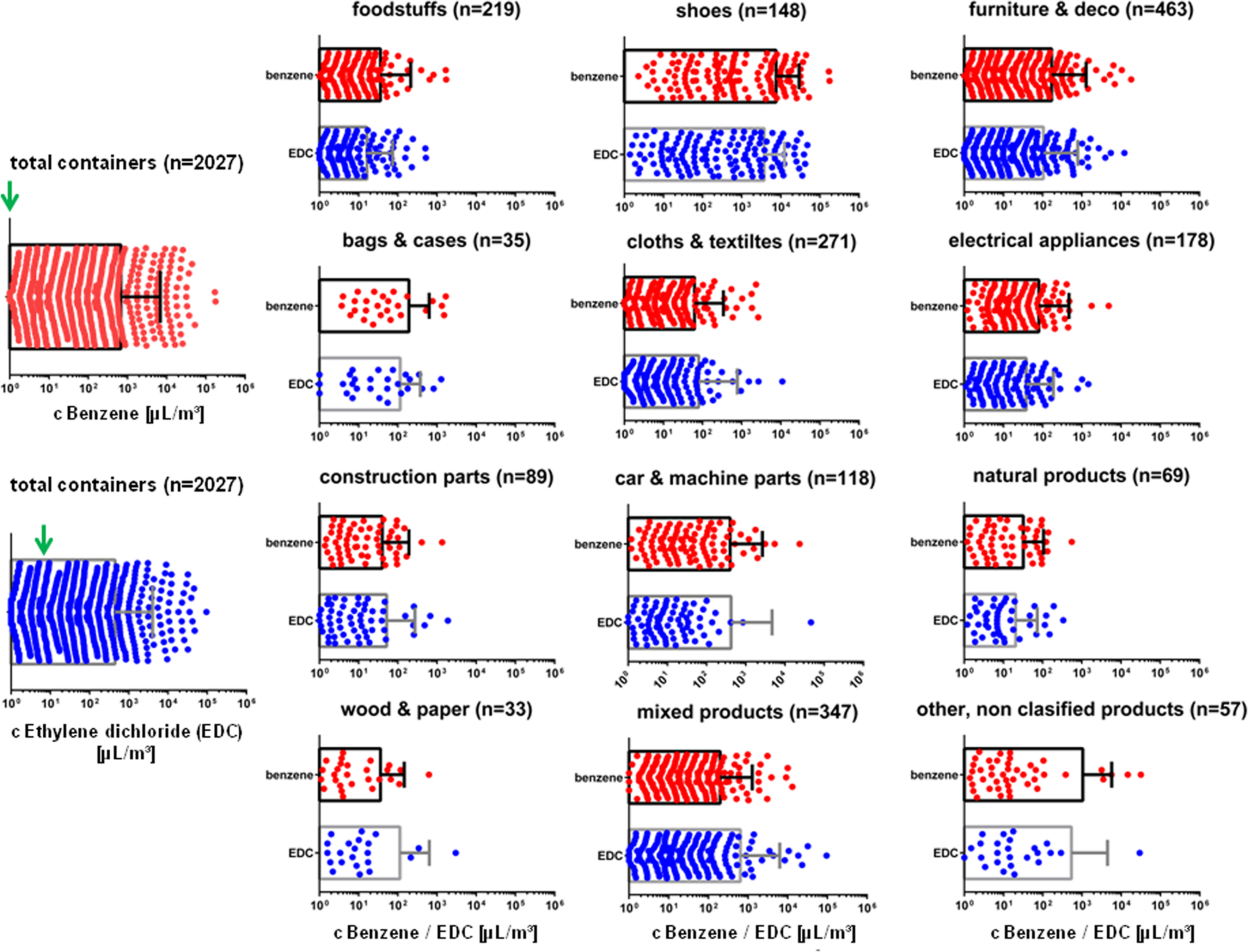
The amounts of the carcinogens benzene and ethylene dichloride detected in container air in total and with respect to the transported product groups. Data show scatter plots with bars (mean ±SD). To make the data more visible and comparable all axes were set in the range between 10^0^ **μ**L/m^3^ppb (10 ppb) and 10^6^ **μ**L/m^3^. The Green arrows show RELs, recommended exposure limit.

To evaluate the levels of presumably carcinogenic chemicals in containers in correlation with the transported products, we have first analyzed the container air in 2,027 randomly chosen containers arriving at the harbor of Hamburg in the years 2010–2014 by using TD-GC/MS[29]. We then evaluated gas concentrations in the container air and calculated the numbers of transport units with the gas atmosphere higher than the community relevant health based exposure levels (RELs). Previous data have shown that contamination with industrial chemicals appear to provide a greater problem as the fumigants themselves [10, 12]. We have now focused mainly on the carcinogens benzene and ethylene dichloride. The analyzed container air was contaminated with both benzene (mean level: 685 μL/m^3^ ± 139 SEM) and ethylene dichloride (mean level: 447 μL/m^3^ ± 80 SEM) residues (Fig 1). The median values for benzene were much higher than the REL values of 0.98 μL/m^3^ (median = 7.6 μL/m^3^; 95% CI: 6.7, 8.4). The highest maximal concentration found, i.e., 177,158 μL/m^3^, was disturbing, since it exceeded the REL by 180,000-fold. Conversely, for the concentration of ethylene dichloride (EDC) the median values found were not higher than the health-based limit value of 98 μL/m^3^ (median = 3.9 μL/m^3^; 95% CI: 3.4, 4.7). However, the maximal ethylene dichloride concentration was as high as 95,650 μL/m^3^, thus exceeding the REL nearly 1000-fold. The bars in Fig 1 show the measured concentration levels of the different substances (mean ± SD values) within the different product groups investigated. For a better general comparative view these figures only contain the positive values within the same range of log scales and neglect results below the limits of detection.

Further classifications show the amount of ethylene dichloride and benzene concentration in container air grouped by transported goods categories (Fig 1). In all product groups there are containers that show concentrations of ethylene dichloride up to a range of 10–100 times the limit value or higher. Three groups, namely containers transporting cars and vehicle parts, furniture and household goods and shoes reach up to levels more than 1000 times the limit value. While for all other groups the majority of containers (50–75%) remain below the limit value, the majority of containers transporting shoes were found with concentrations above the limit value. 75% of these containers exceeded the limit value for ethylene dichloride, 50% are higher than 10 times the limit value and 25% show concentrations of more than 100 times the limit value. Ethylene dichloride burden of containers transporting bags and accessories is higher than average with more than 50% beyond the limit value. Also for benzene the limit value was exceeded in every commodity group. Again it was the group of containers transporting shoes that revealed most units with high toxicant burden. In this group also the highest benzene concentrations were monitored.

**Fig 2:**
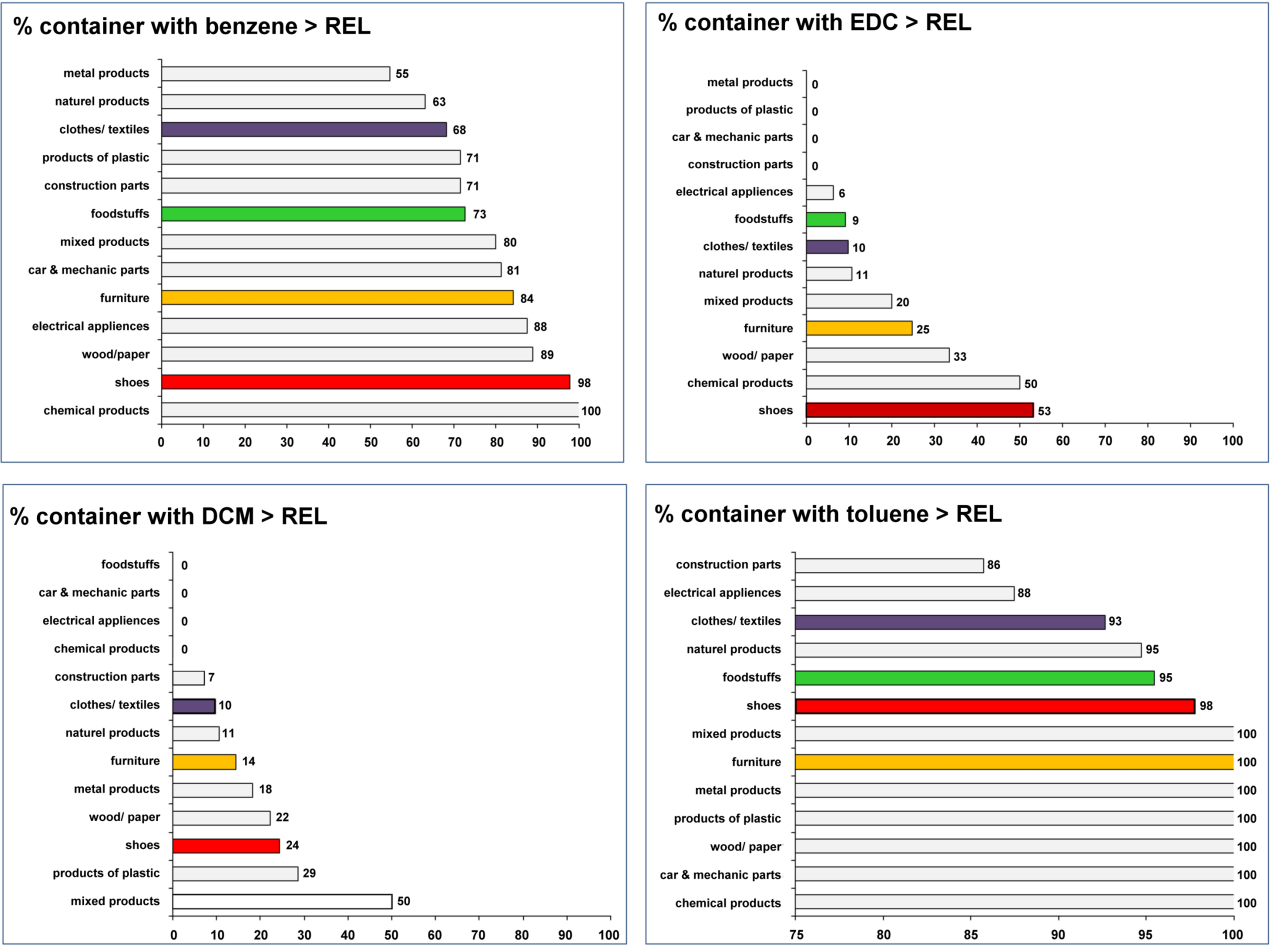
Percentage of the container with industrial chemicals at concentrations higher than the corresponding RELs. RELs within the product group indicated (N-value = 100%, see Fig 1). Highlighted (in purple, green, yellow and red) are products relevant for private consumers. Abbreviations used: EDC, ethylene dichloride; DCM, methylene chloride; REL, recommended exposure limit.

For a better overview, we converted the measured concentrations in container *vs* products to percentage of container showing values higher as the corresponding RELs, as health-based community exposure values (Fig 2). For this overview, we have included not only the carcinogens benzene and ethylene dichloride, but also toluene and dichloromethane. We found that 98% of all containers, which transported shoes, had benzene air concentration higher than the corresponding REL (0.98 **μ**L/m^3^). 53% of shoe containers, 33% of the containers transporting wood/paper, 25% of furniture and 9% of foodstuffs containers were contaminated with ethylene dichloride higher than its REL (>98 **μ**L/m^3^). Containers transporting metal products, car & mechanic parts or construction products had no ethylene dichloride residues that exceeded the REL. Methylene chloride (dichloromethane) amounts above its REL (115 **μ**L/m^3^) were found mainly in containers with mixed products (50%) or plastic products (29%), followed by wood/paper (22%), metal products (18%) and natural products (14%). Conversely, methylene chloride has not been found in containers transporting foodstuff items. More than 90% of all containers revealed toluene residues higher than the health-based community exposure level of 80 **μ**L/m^3^. Only containers transporting electrical appliances or construction products had less toluene (88%, 86%).

For comparison, we analyzed the amounts of classical fumigant residues (methyl bromide, phosphine) in container air (data not shown). Looking for fumigant residues within the individual container groups, 42% of the container units transporting natural products, 36% of containers with construction products, 31% of foodstuff containers, 27% of containers transporting furniture and 17% of the textile/cloth containers had methyl bromide residues higher than its corresponding REL (1 μL/m^3^). None of the containers transporting chemical products had any methyl bromide residues (all were <LOD) and in containers transporting other products (wood, paper, metal or plastic products), including shoe containers, the methyl bromide residues were higher than the REL in only 10% of all cases [9–14%]. Since phosphine was found only in 1% of the randomly selected containers, we did not analyze the distribution of this fumigant within the product groups.

### Contaminated products outgas chemical residues for several days

**Fig 3:**
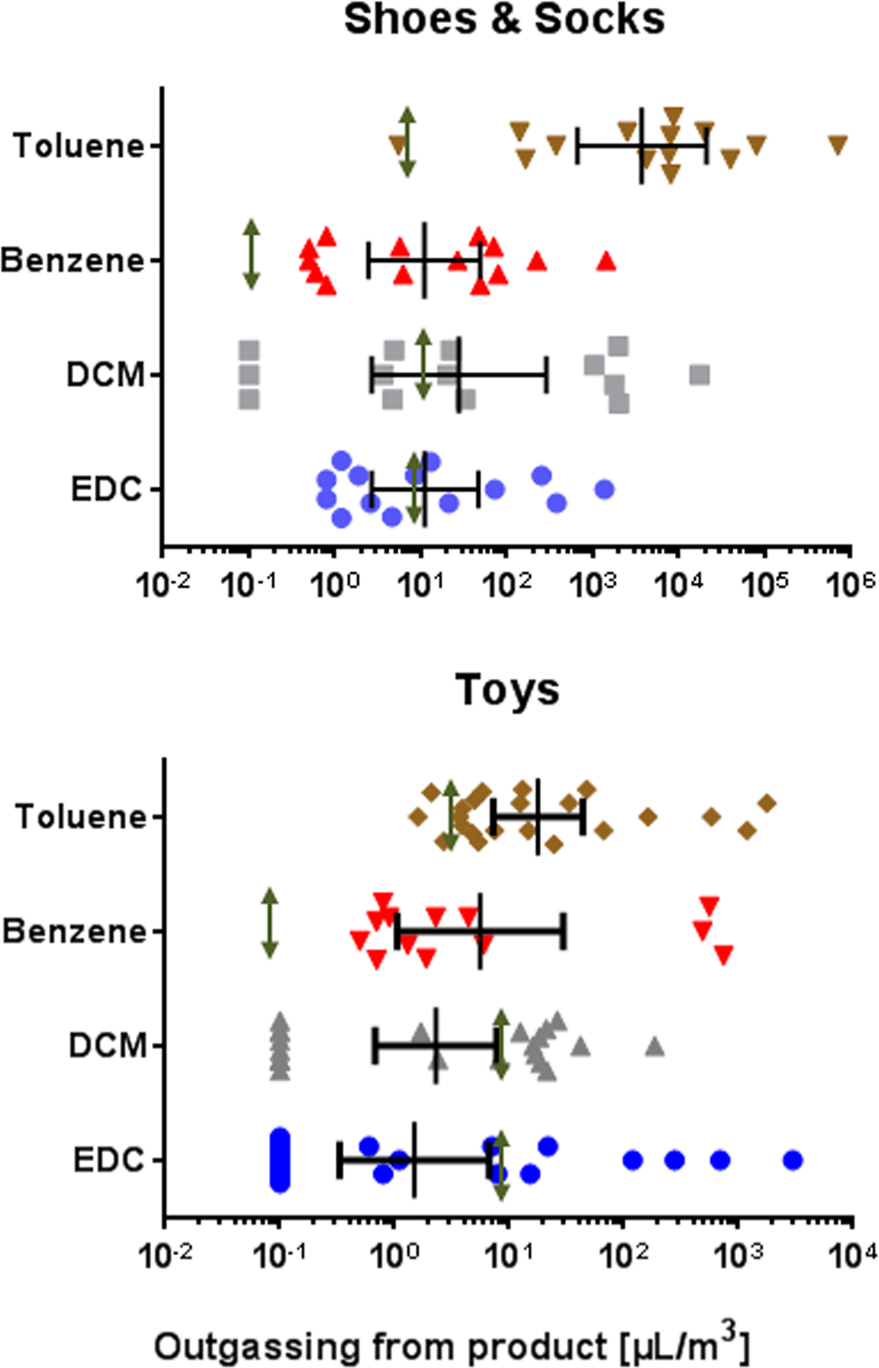
Chemicals outgassing from products taken out from contaminated containers. Children toys (n = 23) and small children shoes plus socks (n = 15) were taken out of the contaminated container and placed in an emission chamber for 24 h. The amounts of outgassing industrial chemicals (toluene, benzene, methylene chloride and ethylene dichloride) were measured as described in the section materials and methods. The lines show geometric mean with 95% CI. The respective REL values are indicated as green arrow lines. Abbreviations used: EDC, ethylene dichloride (1,2-dichloroethane); DCM, methylene chloride (dichloromethane).

We then took several highly contaminated products out of the containers, thereby emphasizing on toys, shoes and socks, assuming that these products, if contaminated, may have the greatest health impact on vulnerable groups such as children. The products (n = 38, note that the sampling was not randomized since we deliberately took products from containers with suspected high volatile concentrations in the air) were placed in an evaporating chamber at room temperature, followed by analysis of the residual outgassing chemicals. The data (Fig 3) show that after one day out of the vested groups, the products were still outgassing benzene and toluene in concentrations higher than the corresponding RELs. More than 50% were outgassing ethylene dichloride and dichloromethane. We decided to let the products outgas for a significant longer time. We took two products, a pair of children`s shoes and a dolls playhouse, contaminated simultaneously with both toluene and ethylene dichloride and monitored the outgassing behaviour for several days. We transferred the items into an emission test chamber. The pair of children’s shoes emitted 115,475 **μ**L/m^3^ toluene, 17,920 **μ**L/m^3^ ethylene dichloride, 1,436 benzene **μ**L/m^3^ and 250 **μ**L/m^3^ methylene chloride at day 1, and was still outgassing levels of 4,194 **μ**L/m^3^ toluene, 47 **μ**L/m^3^ benzene and 32 **μ**L/m^3^ ethylene dichloride after 14 days in the emission chamber. Another product analyzed was a dolls playhouse taken from a container contaminated with ethylene dichloride (45,818 **μ**L/m^3^), toluene (650 **μ**L/m^3^) and benzene (703 **μ**L/m^3^). After 7 days the toy was still outgassing 253 **μ**L/m^3^ toluene, 173 **μ**L/m^3^ benzene and 17,990 **μ**L/m^3^ ethylene dichloride; 21 days later the toy was emitting 5,639 **μ**L/m^3^ ethylene dichloride (a level 5 times higher than the Occupational Exposure Limit, and 57-fold higher than the corresponding REL value) and 15 **μ**L/m^3^ benzene.

### Experimental outgassing of fumigated products

**Fig 4:**
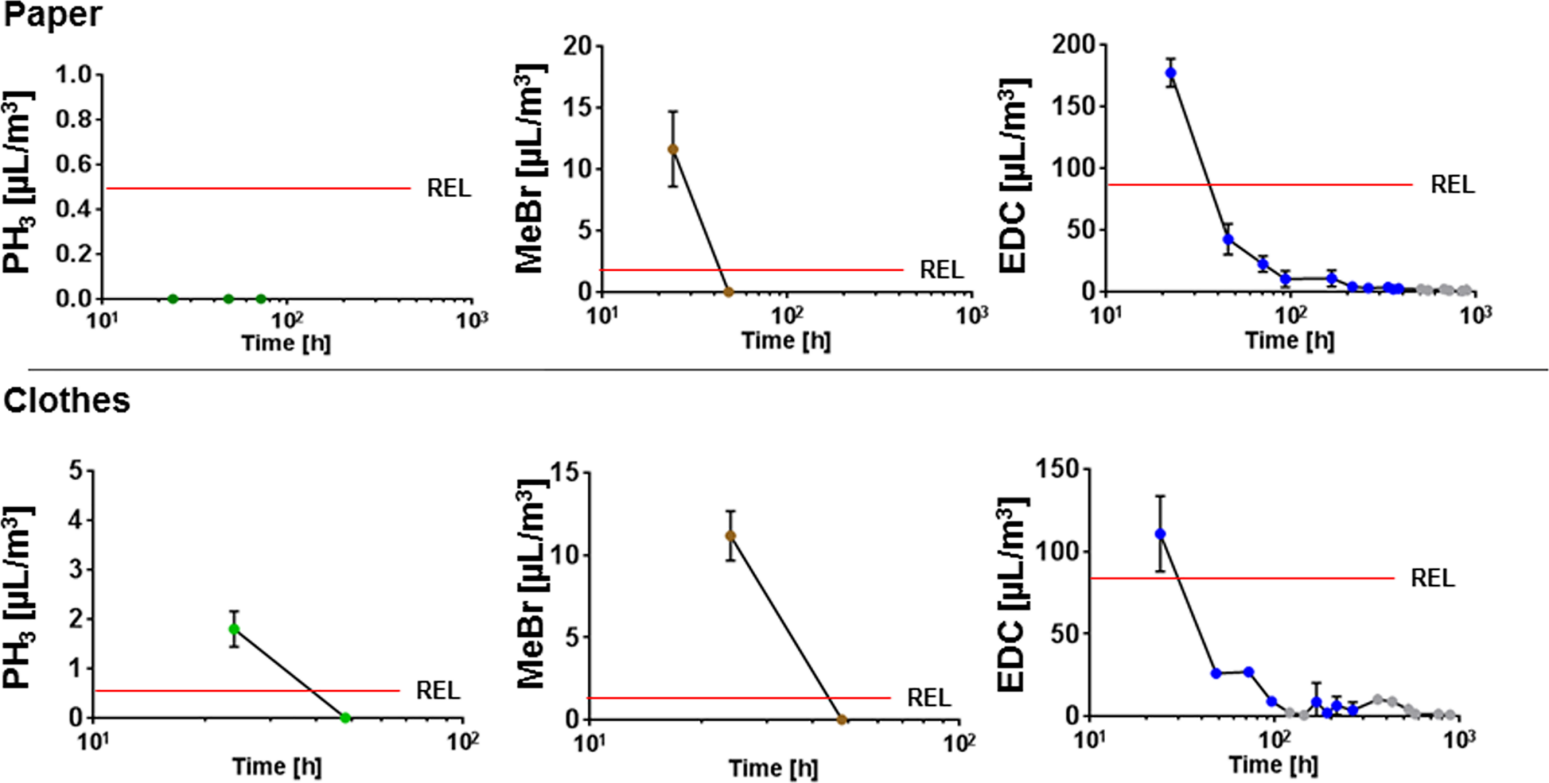
Experimental outgassing of fumigants from products. Two different products from the product groups of packing material and cloths were fumigated with either phosphine, methyl bromide or ethylene dichloride under controlled experimental laboratory conditions (see material and methods). The time-dependently released amounts of trapped and adsorbed gas were measured by TD-GC/MS. Each experiment was repeated three times. Data points show mean values ± SD. Grey dots show analyses below the REL value, which were repeated twice only Abbreviations used: EDC, ethylene dichloride; MeBr, methyl bromide.

We first fumigated the chosen products with the classical fumigants methyl bromide and phosphine (Fig 4, left and middle panels). The amounts of both phosphine (Fig 4, green, left panel) and methyl bromide (Fig 4, brown, middle panel) emitting from fumigated socks decreased below limit values in the course of 48 h. By contrast, the packaging material fumigated with phosphine was still outgassing after 1 day, whereas paper fumigated with methyl bromide was outgassing for 1 day only (Fig 4). Unlike the other fumigants, ethylene dichloride was outgassing from the products for a longer time period (Fig 4, right panel, blue). After 37 days (887 h) and 43 days (1028 h) the concentrations of ethylene dichloride in the collected air samples from outgassing socks and wrapping paper, respectively, were reaching the detection limit.

When assessing the different experimentally fumigated products by using ToF-SIMS, it became clear that the fumigation itself did not alter the structure characteristics of the analyzed textiles or packing materials (data not shown). The fumigants adsorbed on the surface of the product, without undergoing any further chemical interaction with the respective material.

## Discussion

Our data confirm previous studies showing chemical residues in product-transporting containers from us[12, 29] and others[10, 14]. Since only little experimental data is available yet, actually too little to enable any risk assessment for those individuals dealing with contaminated products (like transport or shop workers, by-standers or private consumers), we have performed experimental fumigation and monitored the outgassing time for several specific chemicals and various product groups. Our study shows that unlike products contaminated with classic fumigants (phosphine and methyl bromide) which outgassed more rapidly, the products tainted with industrial chemicals like ethylene dichloride were still outgassing this compound even after 1.5 months. We assume that within the indicated time period the products have reached their destination in storage, production area (i.e. for construction parts or goods to sell), and in private homes from end-consumers. Chemical agents with health hazard or carcinogenic potential and to which storage workers or consumers are likely to be exposed are toluene, benzene, methylene chloride and ethylene dichloride. All of these compounds belong to a group of organic solvents causing potential occupational and home exposure. The source of these chemicals in import containers and related transported goods is mostly unknown. One possibility is their presence in fumigant formulations; they may be residues from container cleaning processes as well as from product outgassing after the manufacturing process. Ethylene dichloride and methylene chloride were used as pesticide fumigants in South America in the past[5]; or as solvents for resins and fats and as gasoline additives to remove lead; they were also used as chemical intermediates in organic synthesis (e.g for vinyl chloride), as extraction solvents and as precursors for cleaning agents for containers. Toluene and benzene can be used in solvent mixtures, cleaning agents and as intermediates in organic synthesis, or being a part of glue or smear. We observed that the amounts of benzene, toluene, and ethylene dichloride varied in individual containers depending on the transported items. Notably nearly 100% of all shoe containers had benzene and toluene levels exceeding the respective RELs, 53% had higher ethylene dichloride levels and 23% higher methylene chloride levels. No methylene chloride higher than RELs was found in foodstuff containers and <10% of these containers had ethylene dichloride levels above the corresponding REL. 73% of these containers were found with measurable benzene concentrations in the air. Although the reference levels these data refer to are quite low, it is important to note that individual transport units had very high concentrations of benzene, toluene, ethylene dichloride or methylene dichloride. Here we show for the first time that products taken out from contaminated containers were still outgassing these volatile industrial chemicals for several weeks, thus being well in the time window in which the products or production parts reach the end-users or public homes. Although we were not able to detect any impact of the chemical contamination on the tested product surface or its properties yet, further studies are required to address this issue more precisely.

It is known that exposure to volatile chemicals can contribute to a wide range of acute and chronic health effects (like asthma, respiratory diseases, liver and kidney dysfunction, neurological impairment and cancer), however exposure related diseases are difficult to detect in non-occupational settings[23, 27, 28, 31]. References on potential health effects mostly result from occupational exposures or experimental data with laboratory animals. Several studies have been published in which the disease or tumor response of animals exposed to solvents/industrial chemicals have been measured. Some recent animal experimental data were concentrating on the adverse effects of sub-acute doses (100–1000 mg/m^3^) of ethylene dichloride showing changes in mice behavior with reduced loco motor and exploratory activities and increased anxiety[32], or on low doses of benzene inducing genotoxic effects[33]. On the other hand, epidemiological data from cohort studies (Weisel 2002) provide credible, but limited evidence that exposures to low dose solvents/industrial chemicals (such as benzene, toluene, ethylene dichloride, methyl chloride) would significantly contribute to the development of chronic diseases and cancer on the basis of cohort studies[27]. The health risk assessment of ambient air concentrations of benzene and toluene has been carried out in service station environments, showing as expected the highest health risk after chronic exposure to the carcinogen benzene[34]. The literature references rely mostly on short term high exposure levels, and focus mostly only on one chemical, thus leaving cumulative or additive effects unreported. Some of the recent epidemiological data, for instance, obtained from occupationally exposed mothers (Infante-Rivard et al. 2005), is more an exception than the rule. In this study of Infante-Rivard and coworkers, an expert exposure assessment method adjusted to low dose occupational solvent mixtures finally allowed to correlate low dose parental exposures with the occurrence of childhood leukemia[35]. Similarly, another recent study [36] has shown an association between resident exposure to solvents and childhood leukemia. Three large population-based case control studies confirmed an increased incidence (OR 1.5–2.2) for non-Hodgkin lymphoma and breast cancer risk following exposure to methylene chloride[19, 37]. Although limited to a small number of studies a comprehensive meta-regression analysis of 9 heterogenic studies [38] revealed a significantly increased risk of leukemia (RR = 1.14, 95% CI 1.04–1.26) at exposure levels as low as 10 ppm-benzene-years. As for many other exposure related diseases, individual risk levels for various mixed exposures and the risk of developing leukemia remain largely unclear. Assuming every day exposures (365 days) one could expect an increased risk for the occurrence of leukemia at levels of benzene as low as 27 ppb (**μ**L/m^3^) benzene per day. No WHO Air Quality Guideline values are available for benzene. However, for this carcinogen the European Air Quality Guideline recommends outdoor exposure levels as low as 5 **μ**g/m^3^ for the annual mean[39].

## Conclusions

The European WHO office recommended a better source control to reduce the indoor concentrations of VOCs[39]. Our data provide evidence that globally produced transported products tainted with toxic industrial chemicals may contribute to the mixture of VOCs in indoor air as they are likely to emit for longer periods than generally anticipated. Children playing on the floor (or crib), ill and elderly persons in poorly ventilated areas are more vulnerable to such emissions[40]. Based on the findings reported we suggest to evaluate the outgassing potency of globally transported products and production parts further. In addition, it seems advisable to address environmental low dose exposure scenarios more carefully in future epidemiological research by applying adequate exposure assessments.

## Abreviations

REL: recommended exposure limits (released by the US Office of Environmental Health Hazard Assessment, OEHHA)
VICs/VOCs: volatile inorganic and organic compounds
EDC: ethylene dichloride (1,2 dichloroethane)
DCM: methylene chloride (dichloromethane)
MeBr: methyl bromide (bromomethane)
FAO: UN Food and Agriculture Organization
WHO: Word Health Organization
